# Understanding creatine for neurological health in babies (UNICORN): an observational cohort study

**DOI:** 10.1080/15502783.2025.2533665

**Published:** 2025-07-19

**Authors:** Nhi T. Tran, Mary J. Berry, Melissa Schlegel, Alison Sheppard, Damien L. Callahan, Miranda L. Davies-Tuck, Natalie Holowko, Rod W. Hunt, David W. Walker, Rod J. Snow, Stacey J. Ellery

**Affiliations:** a The Ritchie Centre, Hudson Institute of Medical Research, Melbourne, Australia; b Department of Obstetrics & Gynaecology, Monash University, Melbourne, Australia; c Department Paediatrics & Child Health, University of Otago, Wellington, New Zealand; d Capital and Coast District Health Board, Wellington, New Zealand; e Centre for Cellular and Molecular Biology, School of Life and Environmental Science, Deakin University, Melbourne, Australia; f Department of Paediatrics, Monash University, Melbourne, Australia; g Monash Children’s Hospital, Melbourne, Australia; h Monash Women’s, Monash Health, Melbourne, Australia; i Institute for Physical Activity & Nutrition, Deakin University, Melbourne, Australia

**Keywords:** Creatine, infant, premature, magnetic resonance spectroscopy, neurodevelopmental disorders, nutritional status

## Abstract

**Background:**

Creatine is essential for brain development. UNICORN is an observational study designed to assess creatine levels in preterm infants, examine creatine availability through nutrition in the weeks after preterm birth, and correlate preterm creatine levels with neurological outcomes.

**Methods:**

Infants were recruited at CCDHB in Wellington, New Zealand. Cord blood samples were collected at birth. Serial blood, urine and nutrition samples were collected for preterm babies between birth and hospital discharge. Creatine concentrations were measured using liquid chromatography-tandem mass spectrometry (LC-MS/MS). A subset of babies underwent a brain magnetic resonance imaging/proton magnetic resonance spectroscopy (MRI/^1^H-MRS) at term corrected age (CA) and neurodevelopmental evaluation with a general movements assessment at three months CA.

**Results:**

Sixty-seven babies (extremely preterm >28 weeks gestational age (GA), n=28; very preterm 28-32 weeks, n=15; moderate-late preterm 32-37 weeks, n=11 and term controls <37 weeks, n=13) were enrolled in the study. Venous cord blood creatine concentrations were not affected by gestational age at birth (p=0.3). However, blood creatine concentrations declined with advancing postnatal age (β -0.77μmol/L, 95% CI -1.05, -0.49; p<0.001). By hospital discharge, blood creatine concentrations of extremely preterm infants were 45% lower than moderate-late and very preterm infants (p=0.03). Infants exclusively fed with total parenteral nutrition (TPN) received no creatine through their diet (TPN creatine 0.8 ± 1.62 μmol/L), compared to those on breastmilk (66.4 ± 29.1 μmol/L) and formula (57.3 ± 30.4 μmol/L).

**Conclusions:**

Analyses are ongoing to assess relationships between an infant’s creatine profile and neurological outcomes. Including creatine supplements in the nutritional management of extremely preterm infants may be beneficial.

